# Supplementary material for: Content-rich biological network constructed by mining PubMed abstracts
Source: BMC Bioinformatics. 2004 Oct 8;5:147. doi: 10.1186/1471-2105-5-147 (PMC528731; doi:10.1186/1471-2105-5-147)
Supplement: Additional File 5 — The original Chilibot query results of the term "long-term potentiation (LTP)" and 22 other terms, limiting the latest references analyzed to the years 1990, 1995, 2000, and 2004. [file 1471-2105-5-147-S5.bz2 › chilibotAdditionalFile5/ltp2004/html/PKC_AMPA.html]

 


 **PKC** and **AMPA** 
  
Found 85 abstracts in PubMed,  **30 abstracts were retrieved and analyzed**.  


---

 Search Google  |
 PDF files only 
|  EDU domain only 

---

**Interactive relationship** (e.g. stimulation, inhibition, etc)

- Cerebellar LTD requires brief activation of  **PKC**  and is expressed as a functional downregulation of  **AMPA**  receptors.  Ref: 12495628 Neuron, 2002
- Additionally,  **AMPA**  receptor activation with kainate in cultured chick amacrine cells leads to translocation of conventional and novel  **PKC**  isoforms to the cell membrane, suggesting that  **PKC**  could be activated upon  **AMPA**  receptor stimulation in these cells.  Ref: 11876774 Eur J Neurosci, 2002
- Cerebellar LTD requires activation of  **PKC**  and is expressed, at least in part, as postsynaptic  **AMPA**  receptor internalization.  Ref: 11144359 Neuron, 2000
- **PKC**  activation induced a simultaneous translocation of calcium calmodulin dependent kinase II CaMKII to synapses but no change in spine number, presynaptic terminal number, or the distribution of  **AMPA**  receptors or the synaptic scaffolding protein PSD 95.  Ref: 11896155 J Neurosci, 2002
- constitutive  **PKC**  activity is necessary for basal  **AMPA**  receptor activity Carvalho et al.  Ref: 11876774 Eur J Neurosci, 2002
- We have previously reported that the activity of alpha amino 3 hydroxy 5 methyl 4 isoxazole propionate  **AMPA**  receptors is potentiated by protein kinase C  **PKC**  in cultured chick retina amacrine neurons,.  Ref: 11876774 Eur J Neurosci, 2002
- Synaptic  **AMPA**  receptor clusters in dendritic spines of Purkinje cells are disrupted upon protein kinase C  **PKC**  mediated phosphorylation of serine 880 in the C terminal domain of GluR2.  Ref: 11248365 Neurosci Res, 2001
- Our results suggest that  **PKC**  gamma binds directly to GluR4, thereby modulating the function of GluR4 containing  **AMPA**  receptors.  Ref: 12471040 J Biol Chem, 2003
- At the postsynaptic membrane of glutamatergic synapses, the cAMP dependent protein kinase PKA, protein kinase C  **PKC** , and calcineurin CaN anchoring protein AKAP79 150 is recruited to NMDA and  **AMPA**  glutamate receptors by postsynaptic density PSD 95 family membrane associated guanylate kinase MAGUK scaffold proteins.  Ref: 12177200 J Neurosci, 2002
- The glutamate receptor subunit 2 GluR2 PDZ ligand domain can be phosphorylated at serine 880 S880, and this disrupts interactions with GRIP ABP glutamate receptor interacting protein  **AMPA**  binding protein but not with PICK1  **PKC**  interacting protein 1.  Ref: 14534256 J Neurosci, 2003
- Cerebellar long term depression LTD is a model of synaptic memory that requires protein kinase C  **PKC**  activation and is expressed as a reduction in the number of postsynaptic alpha amino 3 hydroxy 5 methyl 4 isoxazolepropionate  **AMPA**  receptors.  Ref: 12805550 Science, 2003

**Parallel relationship** (e.g. studied together, co-existance, homology, etc.)

- The results suggest that an increase in  **PKC**  mediated signaling, including, in part, phosphorylation of  **AMPA**  receptors, on striatal spiny neurons may be sufficient to promote the initial appearance... of the levodopa induced motor response changes occurring in a rodent model of the human motor complication syndrome.  Ref: 12691833 Brain Res, 2003
- We conclude that GluR4 is phosphorylated upon  **PKC**  activation and or stimulation of  **AMPA**  receptors in cultured amacrine cells.  Ref: 11876774 Eur J Neurosci, 2002
- In this study, we evaluated the phosphorylation of the GluR4 subunit, which is very abundant in cultured amacrine neurons, to correlate it with the effects of  **PKC**  on  **AMPA**  receptor activity in these cells.  Ref: 11876774 Eur J Neurosci, 2002
- In the present study we investigated the association of protein kinase C  **PKC**  gamma isoform with the GluR4  **AMPA**  receptor subunit.  Ref: 12471040 J Biol Chem, 2003
- The present project was designed to investigate the role of protein kinase A PKA and protein kinase C  **PKC**  in the regulation of phosphorylation of the GluR1 subunits of  **AMPA**  receptors in the spinal cord of rats after capsaicin injection.  Ref: 14559367 Brain Res Mol Brain Res, 2003
- In the present study we have examined the role of  **PKC**  in tetanus induced LTP of  **AMPA**  receptor mediated synaptic transmission in hippocampal slices obtained from adult rats.  Ref: 11069602 Eur J Neurosci, 2000
- Our results suggest that increased phosphorylation of the GluR1 subunit of  **AMPA**  receptors contributes to central sensitization following acute peripheral inflammation, and the effect may occur at different phosphorylation sites through the activation of the PKA or  **PKC**  protein kinase cascades.  Ref: 14559367 Brain Res Mol Brain Res, 2003
- Since protein kinase C  **PKC**  mediated phosphorylation regulates glutamatergic receptors of the alpha amino 3 hydroxyl 5 methyl 4 isoxazole propionic acid  **AMPA**  subtype and has been linked to several forms of behavioral plasticity, activation of  **PKC**  signaling in striatal spiny neurons may also contribute to the motor plasticity changes associated with chronic levodopa therapy.  Ref: 12691833 Brain Res, 2003
- PDZ proteins interacting with C terminal GluR2 3 are involved in a  **PKC**  dependent regulation of  **AMPA**  receptors at hippocampal synapses.  Ref: 11163273 Neuron, 2000
- **PKC**  gamma was co immunoprecipitated with GluR4  **AMPA**  receptor subunit in rat cerebellum and in cultured chick retina cell extracts, and immunocytochemistry experiments showed co localization of GluR4 and  **PKC**  gamma in cultured chick retinal neurons.  Ref: 12471040 J Biol Chem, 2003
- Phorbol esters, such as tetradecanoylphorbol 13 acetate TPA, have been used extensively in studies of cerebellar long term depression LTD, based on the hypothesis that activated protein kinase C  **PKC**  directly mediates alpha amino 3 hydroxy 5 methyl 4 isoxazolepropionic acid  **AMPA**  receptor phosphorylation.  Ref: 14529724 Neuropharmacology, 2003
